# Elucidation of the molecular responses to waterlogging in *Sesbania cannabina* roots by transcriptome profiling

**DOI:** 10.1038/s41598-017-07740-5

**Published:** 2017-08-23

**Authors:** Cheng-Gang Ren, Cun-Cui Kong, Kun Yan, Hua Zhang, Yong-Ming Luo, Zhi-Hong Xie

**Affiliations:** 1 Key Laboratory of Biology and Utilization of Biological Resources of Coastal Zone, Yantai Institute of Coastal Zone Research, Chinese Academy of Sciences, Yantai, 264003 China; 2 Key Laboratory of Coastal Environmental Processes and Ecological Remediation, Yantai Institute of Coastal Zone Research, Chinese Academy of Sciences, Yantai, China

## Abstract

*Sesbania cannabina*, a multipurpose leguminous crop, is highly resistant to waterlogging stress. However, the scant genomic resources in the genus *Sesbania* have greatly hindered further exploration of the mechanisms underlying its waterlogging tolerance. Here, the genetic basis of flooding tolerance in *S. cannabina* was examined by transcriptome-wide gene expression changes using RNA-Seq in seedlings exposed to short-term (3 h) and long-term (27 h) waterlogging. After de- novo assembly, 213990 unigenes were identified, of which 145162 (79.6%) were annotated. Gene Ontology and pathway enrichment analyses revealed that the glycolysis and fermentation pathways were stimulated to produce ATP under hypoxic stress conditions. Energy-consuming biosynthetic processes were dramatically repressed by short and long term waterlogging, while amino acid metabolism was greatly induced to maintain ATP levels. The expression pattern of 10 unigenes involved in phenylpropanoid biosynthesis, glycolysis, and amino acid metabolism revealed by qRT-PCR confirmed the RNA-Seq data. The present study is a large-scale assessment of genomic resources of *Sesbania* and provides guidelines for probing the molecular mechanisms underlying *S. cannabina* waterlogging tolerance.

## Introduction

Flooding and waterlogging are common phenomena with the potential to become an increasingly serious threat to the environment, which is progressively deteriorated by humans and their anthropogenic activities, resulting in global climate change. Adverse abiotic stresses can heavily damage crop production worldwide during the heavy rain season in lowland fields. Because of the limited diffusion of gas under water, waterlogging creates a low oxygen (hypoxia) environment in the root areas of crops^1^.

Higher plants are aerobic organisms. Since the diffusion of molecular oxygen is reduced in water, soil waterlogging is a serious obstacle to plant growth and development and may make plants hypoxic. The response of plants to external hypoxia has been intensively studied for decades. Alternative to oxidative respiration, plants employ glycolysis to generate ATP and ethanolic fermentation to produce the NAD^+^ required for sustaining the EMP pathway^2^. Not only metabolic adaptations which confer tolerance to anoxia, prolong tissue survival, there are also morphological adaptations to waterlogging by plants, such as formation of aerenchyma^3^. Understanding the genetic basis of tolerance variation and the underlying genes and processes will be the key to discovering novel tolerance mechanisms and ultimately translating these to crops^4^.

Proteomics research has identified many anaerobically induced polypeptides (ANPs)^5^. ANPs are essential for the tolerance to hypoxic conditions in many plants^6, 7^. Further studies showed that the majority of ANPs are involved in the glycolysis and fermentation pathways^8^. A set of anaerobic peptides including aldolase, enolase, glucose-6-phosphate isomerase, glyceraldehyde-3-phosphate dehydrogenase, sucrose synthase, and alcohol dehydrogenase have been identified as being selectively induced under hypoxia in maize^9^. Microarray studies were performed to analyze the response to hypoxia in *Arabidopsis thaliana* ^10^, cotton^6^, maize^11^, and other plants^12^. The expression levels of a zinc finger-like protein, SKP1/ASK1-like protein, and 20 S proteasome subunit α-3 increased markedly after 2 h of minimal oxygen supply in maize^13^. A low oxygen-sensing N-end rule proteolytic pathway^14^ and a gene, *Sicyp51*, believed to confer tolerance to hypoxia were identified recently^15^. SNORKEL1 and 2 in rice and MYB, and AP2/ERF transcription factors (TFs) in *Arabidopsis* ^16–18^ were found to regulate gene expression under low oxygen. In *Glycine max*, waterlogging stress specific genes were found to be enriched for lipoxygenase 2, ethylene forming enzyme, matrixin family proteins, and 12-oxophytodienoate reductase 2. The Ethylene response factor (ERF) represented the highest number of significantly expressed TFs under hypoxic conditions followed by bHLH, MYB, NAC, and WRKY^19^. Rapid changes in a large number of transcripts involving not only well-known ANPs, but also those not previously known to be involved in waterlogging tolerance, indicate that plants activate complex mechanisms in response to waterlogging^6, 20^.

The RNA-Seq approach has a higher sensitivity than microarray analysis and includes both low- and high- level gene expression^21^. These advantages resulted in the increased application of RNA-Seq to elucidate the response of plants to various environmental stresses, such as cold^22^, salt^23, 24^, and drought^23, 25^. RNA-Seq has also been successfully used to analyze the responses to waterlogging stress in maize^26^, sesame^27^, and rape^28^. However, transcriptional studies of manure crops are rare, despite their potential in environment-friendly agricultural practices.

The genus *Sesbania* has been used increasingly as a green manure crop in rice cropping systems because of its ability to fix atmospheric nitrogen through symbiosis with rhizobia and its adaptability to flooded soils^29–31^. Of approximately 20 species distributed in these regions, *S. cannabina* is one of the most common species, and its ability to promote the drainage of ill-drained fields and paddy-upland rotated fields has been demonstrated^32^. To better understand the molecular mechanisms underlying the response of *S. cannabina* to soil waterlogging, the transcriptional profiles of waterlogged *S. cannabina* roots were analyzed by RNA-Seq. Genes expressed in the control and treatment groups were compared to elucidate the species-specific responses of *S. cannabina* and to identify genes or strategies associated with waterlogging resistance. Our results will improve our understanding of the response of flood-tolerant manure crops to soil waterlogging stress.

## Results

### Transcriptome sequencing and assembly

To obtain a comprehensive view of waterlogging tolerance in *S. cannabina*, 30-day old seedlings of *S. cannabina* were waterlogged. High-throughput RNA sequencing (RNA-Seq) was performed on waterlogged roots (3 h, and 27 h), non- waterlogged control roots (CK) using the Illumina platform. Illumina sequencing data were deposited in the NCBI GEO database under accession number GSE92670. In total, 321,027,022 Illumina PE raw reads were generated (Table 1). After removing adaptor sequences, ambiguous nucleotides, and low-quality sequences, 302,739,278 clean reads were recorded. Assembly of clean reads resulted in 213,990 unigenes in the range of 201–16,929 bp with a N50 length of 1055 bp (Table S1).

**Table 1:** Summary of sequences analysis.

Sample	Raw Reads	Clean Reads	Clean	Error	Q20	Q30	GC
			Bases	(%)	(%)	(%)	(%)
R1	46891272	43971630	6.6G	0.02	96.1	90.69	43.71
R2	48598584	45774804	6.87G	0.02	96.43	91.34	43.98
WLR3H1	51310282	49550926	7.43G	0.01	97.02	92.65	44.37
WLR3H2	64341606	61288682	9.19G	0.02	96.2	90.84	43.89
WLR27H1	55278170	51429496	7.71G	0.03	94.78	87.72	45.29
WLR27H2	54607108	50723740	7.61G	0.03	94.66	87.4	44.91
Total	321027022	302739278	45.41G				

R, Untreated root; WLR, Waterlogged root; H, Hour; Q20, The percentage of bases with a Phred value > 20; Q30, The percentage of bases with a Phred value > 30.

### Sequence annotation

Next, the unigenes were annotated by alignment with seven public databases (Table 2). Analyses showed that 155,725 unigenes (72.77%) had significant matches in the Nr database, 143,553 (67.08%) in the Nt database, and 105,129 (49.12%) in the Pfam database. In total, 170,345 unigenes (79.6%) were successfully annotated in at least one of the Nr, Nt, Swiss-Prot, KEGG, GO, KOG, and Pfam databases, with 1054 unigenes (0.49%) in all seven databases. Most of the unigenes could be mapped to legumes sequences, in which 31,715, 30,692, 28,198 and 21,235 showed high sequence similarity with that of *Glycine max*, *Glycine soja, Cicer arietinum* and *Medicago truncatula*, respectively (Table 3).

**Table 2:** BLAST analysis of non-redundant unigenes against public databases.

	Number of	Percentage
	Unigenes	(%)
Annotated in NR	155725	72.77
Annotated in NT	143553	67.08
Annotated in KO	61329	28.65
Annotated in SwissProt	3772	1.76
Annotated in PFAM	105129	49.12
Annotated in GO	106734	49.87
Annotated in KOG	58137	27.16
Annotated in all Databases	1054	0.49
Annotated in at least one Database	170345	79.6
Total Unigenes	213990	100

**Table 3:** The top-10 Blast hits for the assembled unigenes based on NCBI non-redundant (nr) protein database search.

Species	Number of annotations	Plant_nr
*Glycine max*	31715	63716
*Glycine soja*	30692	50066
*Cicer arietinum*	28198	29056
*Medicago truncatula*	21235	90439
*Phaseolus vulgaris*	16644	32585
*Lotus japonicus*	6093	8640
*Vitis vinifera*	1773	74877
*Gossypium raimondii*	1098	86601
*Citrus sinensis*	1054	66922
*Pyrus x*	784	38836

**Figure 1:**
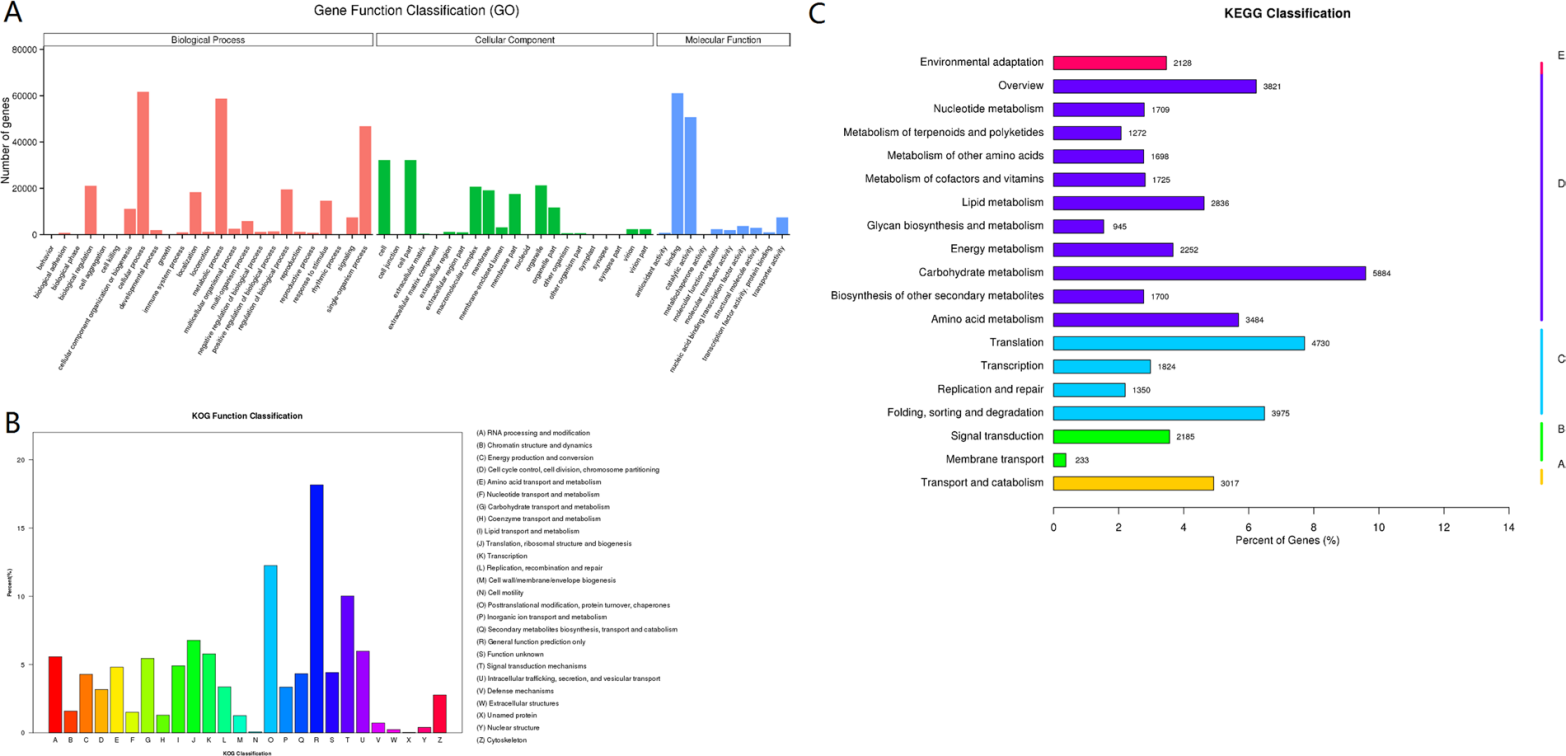
Functional annotation and classfication of *S. cannabina* transcriptome. (**A**) GO categorization of non-redundant unigenes. Each annotated sequence was assigned at least one GO term. (**B**) COG annotation of putative proteins. (**C**) KEGG annotation of putative proteins. The capital letters against the colored bars indicate five main categories, (**A**) cellular processes; (**B**) environmental information processing; (**C**) genetic information processing; (**D**) metabolism; and (**E**) organism systems.

For GO analysis, there were 106,734 unigenes divided into three ontologies (Fig. 1A). For the biological process (BP) category, genes involved in ‘cellular process’ (61,631), ‘metabolic process’ (58,709), and ‘single-organism process’ (46,928) were highly represented. The cellular component (CC) category mainly comprised proteins involved in ‘cell’ (32,127), ‘cell part’ (32,126), and ‘organelle’ (21,218). Within the molecular function (MF) category, ‘binding’ (61,098), ‘catalytic activity’ (50,782), and ‘transporter activity’ (7502) were highly represented.

In addition, all unigenes were subjected to a search against the KOG database for functional prediction and classification. In total, 31,137 unigenes were assigned to the KOG classification and divided into 26 specific categories (Fig. 1B). The ‘general functional prediction only’ (10554) was the largest group, followed by ‘post-translational modification, protein turnover, chaperone’ (7119), ‘signal transduction mechanisms’ (5822), ‘translation, ribosomal structure and biogenesis’ (3935), and ‘intracellular trafficking, secretion, and vesicular transport’ (3471). Only a few unigenes were assigned to ‘cell motility’ (42) and ‘unnamed protein’ (15).

Unigene metabolic pathway analysis was also performed using the KEGG annotation system. This process predicted a total of 130 pathways representing 61,329 unigenes (Fig. 1C). The pathways involving the highest number of unique transcripts were ‘carbohydrate metabolism’ (5884), followed by ‘translation’ (4730) and ‘folding, sorting and degradation’ (3975).

### Differential expression analysis of assembled transcripts in response to waterlogging treatments in the early and late stages

**Figure 2:**
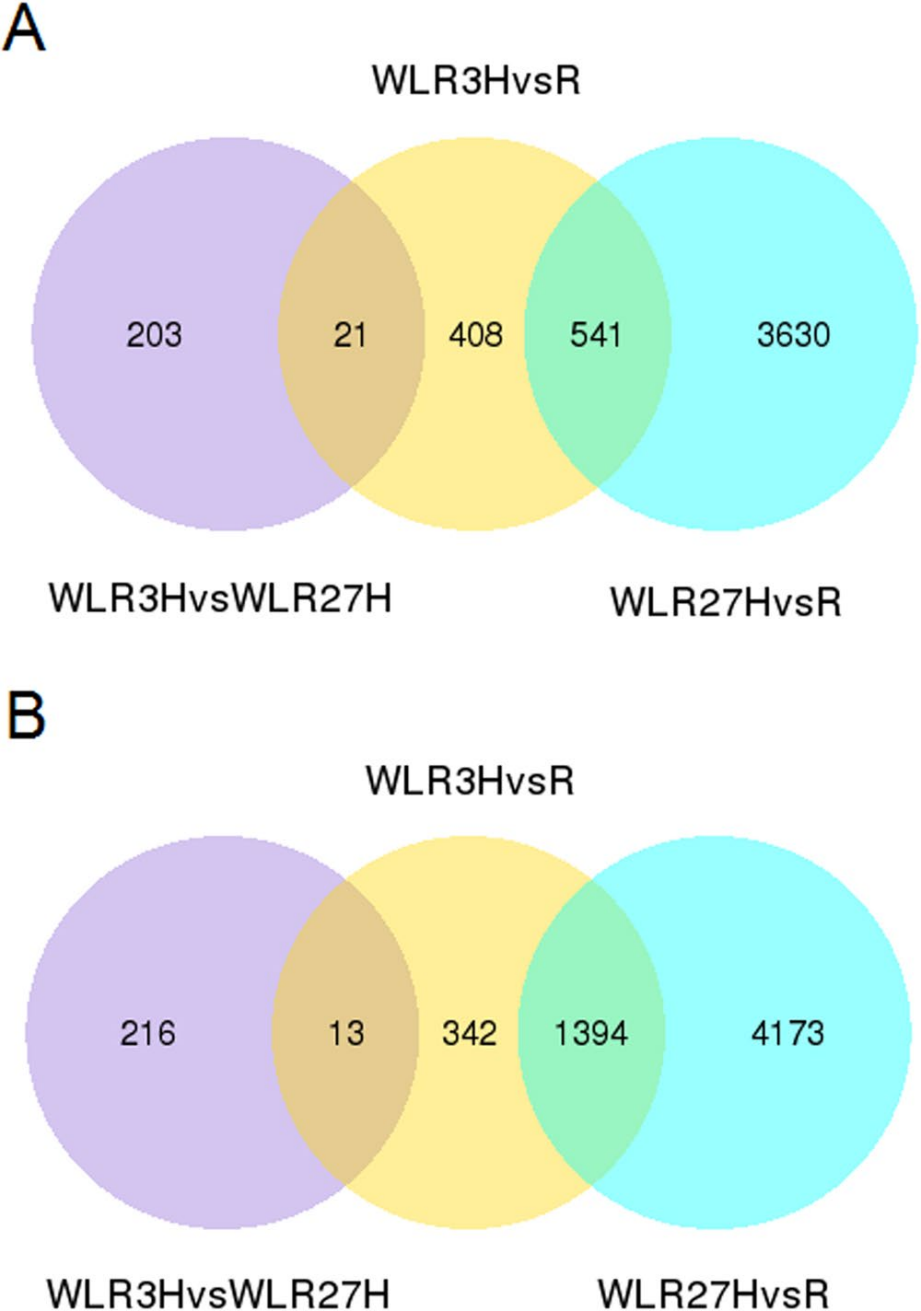
Venn diagrams of differentially expressed transcripts under waterlogging treatment in *S. cannabina* plantlets. (**A**) Numbers of DEGs exclusively upregulated in each treatment; (**B**) numbers of DEGs exclusively downregulated in each treatment. The numbers of DEGs with common or opposite expression change tendencies between treatments are shown in the overlapping regions. The total numbers of up- or down-regulated genes in each treatment are the sum of the numbers in each circle.

Differential expression analysis was firstly performed based on pairwise comparison between different waterlogging treatment times (CK, 3 h and 27 h). DEGs were defined as genes that were significantly enriched or depleted by the treatments. A total of 2719 transcripts, which accounted for approximately 1.27% of the total transcripts were differentially expressed in *S. cannabina* in response to the early stages (3 h) of waterlogging (Table S2). Meanwhile, a total of 9738 transcripts, which accounted for approximately 4.5% of the total transcripts were differentially expressed in *S. cannabina* in response to the latestages (27 h) of waterlogging (Table S2). Commonly up- and down-regulated transcripts were identified between the early and late stages of waterlogging to determine the degree of overlap (Fig. 2A,B). A total of 970 transcripts were upregulated and 1,749 transcripts were downregulated between the CK and 3 h waterlogged groups. The maximum number of commonly up- (4,171) and down- (5,567) regulated transcripts was observed between the CK and 27 h waterlogged groups.

To explore patterns of co-regulation of the DEGs in *S. cannabina* in response to the early and late stages of waterlogging, the expression profiles of these genes were clustered using the hierarchical clustering algorithm and visualized in a heat map (Fig. S1). The results showed distinct patterns of expression of DEGs in response to different stages of waterlogging stress.

### Functional classification of DEGs

**Figure 3:**
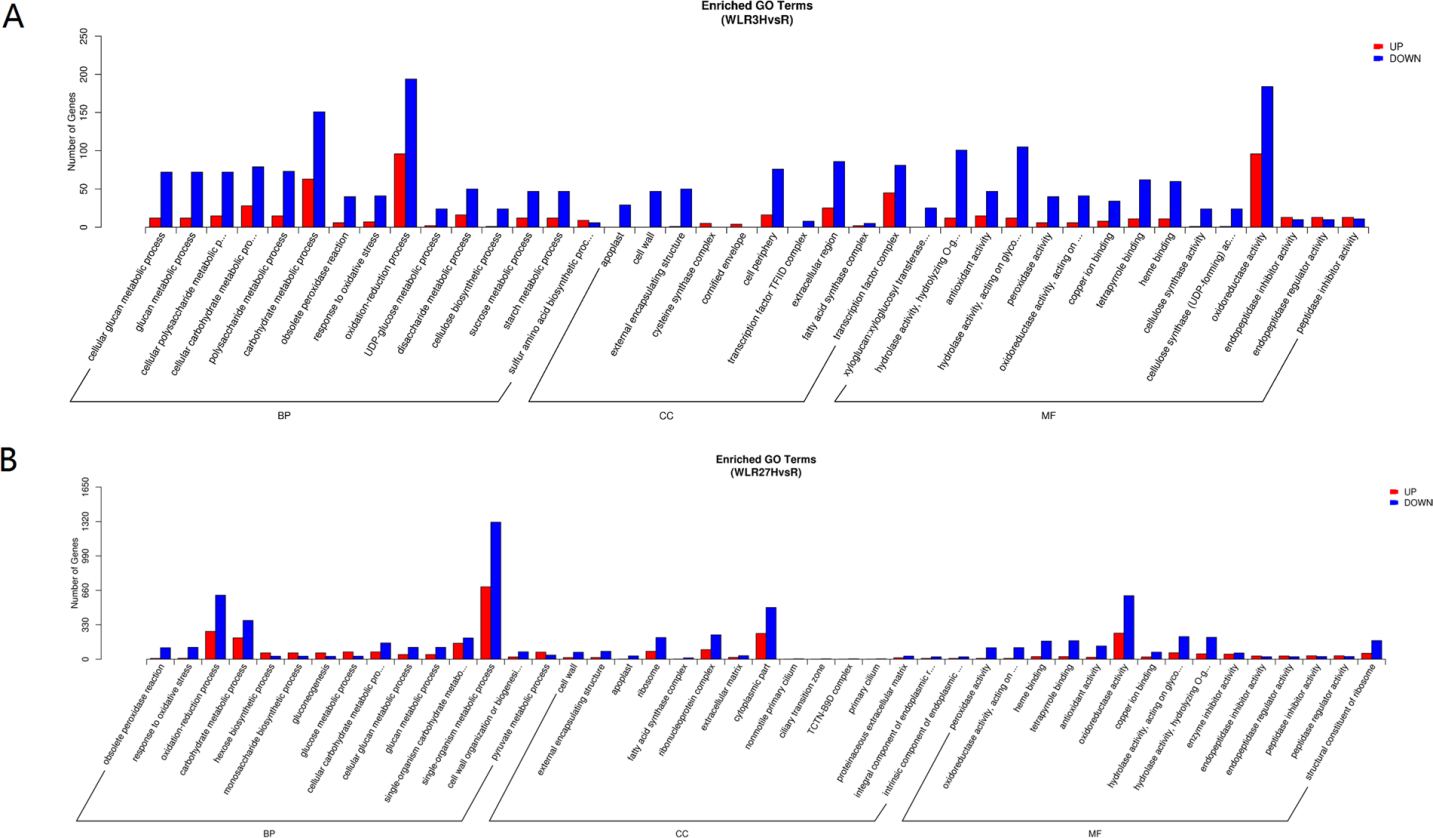
GO functional classification of the control and waterlogging treated plantlets. (**A**) comparison between 3 h treatment and CK; (**B**) comparison between 27 h treatment and CK.

To gain a better understanding of the adaptive mechanism shift in *S. cannabina* in response to waterlogging, biological function analysis of DEGs was performed by GO enrichment using the whole transcriptome as the background (Fig. 3). GO analysis was performed for upregulated DEGs in CK compared with those in the 3 h waterlogging group (Table S3). In the MF category, the top two enriched terms were oxidoreductase activity and transporter activity, followed by enzyme inhibitor activity. In the CC category, ‘integral component of membrane’ and ‘intrinsic component of membrane’ were the two dominant enriched terms. In BP, ‘oxidoreduction coenzyme metabolic process’ was the most enriched, and various terms of metabolic processes were also enriched, including ‘pyridine-containing compound metabolic process’, ‘pyruvate metabolic process’ and ‘glucose metabolic process’ (corrected p-value < 0.005). For the downregulated DEGs (Table S4), the most enriched terms were oxidoreductase activity in the MF category. Consistent with this, ‘oxidation-reduction process’ was the top BP enriched term in downregulated DEGs, which taken together with ‘oxidoreductase activity’ as the most enriched in upregulated DEGs implied that different sets of oxidoreductase genes participate in either maintaining redox equilibrium in a normal environment or in alleviating the oxidative stress caused by waterlogging. Other enriched terms included carbohydrate metabolic process and hydrolase activity, acting on glycosyl bonds.

Comparison of DEGs in 27 h waterlogging with those in CK identified ‘metabolic process’ and ‘catalytic activity’ as the top two GO enrichment terms in the downregulated DEGs (Table S5). These two terms included >2000 DEGs, which were all repressed. Additionally, 100% of DEGs involved in ‘hydrolase activity’, ‘cellulose synthase (UDP-forming) activity’ and ‘cellulose biosynthetic process’ were also downregulated. Changes in transcript levels suggested that the energy-demanding processes of cellulose and cell wall biosynthesis were greatly inhibited in the *S. cannabina* response to the late stage of waterlogging. For the GO enrichment analysis of the upregulated DEGs, ‘transcription factor complex’, ‘glucose metabolic process’ and ‘enzyme regulator activity’ were the dominant terms (Table S6).

KEGG pathway enrichment analysis for DEGs also revealed common patterns and significant differences in response/adaptation between the early and late stages of waterlogging in *S. cannabina*. The top five enriched pathways by DEGs in the 3 h waterlogging treatment (Table S7) (FDR ≤ 0.05), were phenylalanine metabolism, cysteine and methionine metabolism, phenylpropanoid biosynthesis, tyrosine metabolism and sulfur metabolism. DEGs in the 27 h waterlogging treatment was also analyzed (Table S8) (FDR ≤ 0.05). The top two enriched pathways by DEGs in the 27 h waterlogging treatment were biosynthesis of secondary metabolites and phenylpropanoid biosynthesis. Phenylpropanoid biosynthesis was the third and second enriched pathway by DEGs in 3 h and 27 h waterlogged plantlets, respectively. There were 58 and 186 unigenes annotated as involved in this pathway in 3 h and 27 h waterlogged plantlets, respectively, with 45 shared unigenes showing changed expression both in the early and late stage of waterlogging stress (Fig. S2). Ten upregulated DEGs were annotated as encoding enzymes involved in phenylpropanoid biosynthesis. Among them, five DEGs (Cluster-8728.18814, Cluster-8728.82713, Cluster-8728.82715, Cluster-8728.84811, Cluster-8728.109527) were involved in the early stage of waterlogging and six DEGs (Cluster-8728.90349, Cluster-8728.158848, Cluster-8728.18814, Cluster-8728.94869, Cluster-8728.117414, Cluster-8728.94871) were involved in the late stage of waterlogging, with one shared DEG annotated as 4-coumarate–CoA ligase-Cluster-8728.18814 showing 4.75- and 6.54-fold increased expression, respectively (Table S9). Phenylpropanoid biosynthesis is regulated by biotic and abiotic stimuli, and phenylpropanoid-based polymers such as lignin, suberin, and tannin contribute substantially to the stability and robustness of plants in the face of mechanical or environmental damage^33–35^. On the other hand, their downregulation suggested the harmful effects of waterlogging. There were 58 downregulated DEGs in the early stage of waterlogging and 176 downregulated DEGs in the late stage of waterlogging (Fig. S2).

**Figure 4:**
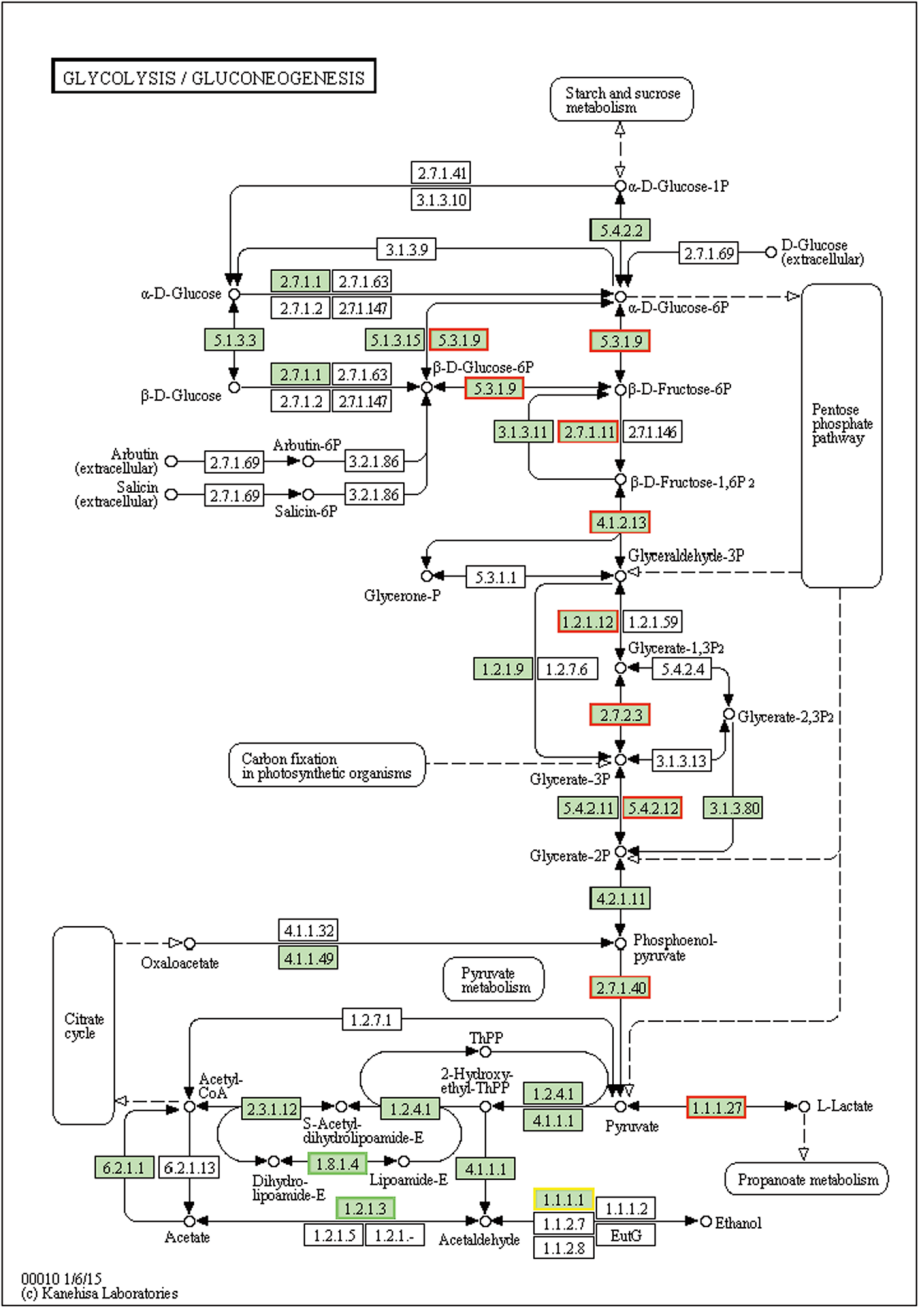
Unigenes predicted to be involved in the glycolysis pathway derived from KEGG database^61^. Red outline indicates significantly increased expression at 3 h compared with 0 h waterlogging treatment; green outline indicates significantly decreased expression; yellow outline indicates proteins encoded by both up-and down-regulated genes.

Most of the ANPs identified were found to be enzymes of glycolysis or sugar-phosphate metabolism^8^. There were 32 unigenes annotated as encoding enzymes involved in glycolysis/gluconeogenesis pathways in the early stage of waterlogging and no unigenes were significantly enriched in the late stage of waterlogging (Fig. 4, Tables S7 and S8). There were five DEGs annotated as encoding glucose-6-phosphate isomerase (GPI): Cluster-8728.113805, Cluster-8728.113801, Cluster-8728.113802, Cluster-8728.120033, and Cluster-8728.112865 had more than 2-fold increased expression. The activities of the corresponding enzymes 6-phosphofructokinase (6-PFK), fructose-bisphosphate aldolase class I (FBA), glyceraldehyde-3-phosphate dehydrogenase (GAPDH), phosphoglycerate mutase (PGAM), and pyruvate kinase (PDC) were in turn upregulated. Supporting our results, lactic acid fermentation is activated during the initial stages of root hypoxia in many plants. In gray poplar, lactate dehydrogenase transcripts increase as an initial reaction to O_2_ deprivation, whereas they decrease after approximately 5 h because of the decrease in cytosolic pH caused by lactic acid^36^. In plants, lactic acid fermentation is usually followed by alcoholic fermentation, which is the major fermentation process for plants. In our results, five upregulated DEGs were annotated as encoding alcohol dehydrogenase (ADH). The activation of alcoholic fermentation resulted in energy production in 3 h waterlogged *Sesbania cannabina* plantlets. However, none of these DEGs showed changed expression in 27 h waterlogged plantlets.

**Figure 5:**
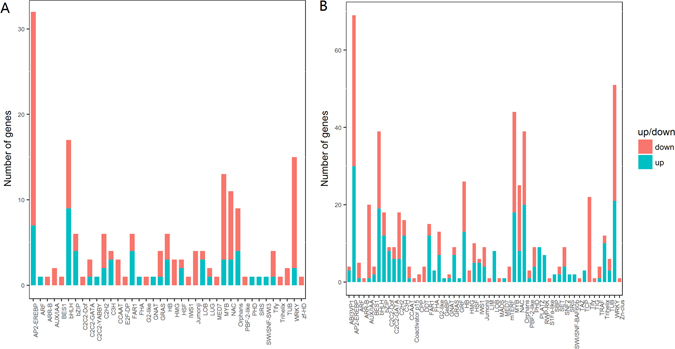
Class-wise counts of transcription factors identified in DEGs under waterlogging. (**A**) TFs (up- and down-regulated) were found in 3 h waterlogging. (**B**) TFs (up- and down-regulated) were found in 27 h waterlogging.

Since low oxygen induced TFs could mediate the expression of anaerobic responsive genes, we analyzed for TFs that were differentially regulated by both early and late stages of waterlogging. Total 176 waterlogging-regulated TFs were classified based on their assigned protein families in early stage, counted for 6.1% of the DEGs. Of these, 60 TFs were up-regulated and 116 TFs were downregulated (Fig. 5A; Table S10). Total 593 waterlogging-regulated TFs were classified in late stage, counted for 6.1% of the DEGs. Among them, 286 TFs were up-regulated and 307 TFs were down-regulated (Fig. 5B; Table S10). Genes belonging to the AP2-EREBP, WRKY, MYB and bHLH family represent most of the differentially expressed TFs. The bHLH family represented the highest number of significantly expressed TF in early stage of wterlogging, while ethylene response element binding protein (EREBP) represented the highest number of significantly expressed genes in late stage of waterlogging (Fig. 5).

### Verification of RNA-Seq data by real-time quantitative RT-PCR

The differential expression of selected transcripts from RNA-Seq data was validated by real-time PCR analysis. The expression analysis was performed for selected genes belonging to the phenylpropanoid biosynthesis pathway, glycolysis pathway, and amino acid metabolism. In addition, WRKY20 and NAD(P)H dehydrogenase, which showed differential expression, were also selected randomly from the transcriptome dataset and validated by real-time PCR analysis. A comparative analysis of all the selected genes showed that the expression patterns in real-time PCR analysis were similar to those observed in the RNA-Seq data (Fig. S3). The Pearson correlation coefficient was calculated by SPSS to assess the correlation between different platforms (Fig. S4). We observed a good concordance in the expression patterns of DEGs obtained by the RNA-Seq and qRT-PCR methods, as indicated by the correlation coefficient (0.942 and 0.933), confirming that our experimental results were valid.

## Discussion

### Effects on energy-consuming biosynthetic processes

Low oxygen conditions in plants shift metabolism from oxidative phosphorylation to anaerobic fermentation to maintain ATP production^2^. Our results supported the longstanding notion that waterlogging promotes anaerobic respiration, as observed by the upregulation of DEGs encoding enzymes involved in glycolysis and fermentation (Fig. 4). On the other hand, plants can activate additional responses to low oxygen conditions, including the downregulation of energy consuming processes. In the present study, genes involved in the biosynthesis of carotenoids, flavonoids, phenylpropanoids, and fatty acid elongation were downregulated in the roots of waterlogged *S. cannabina* (Table S9). These results, together with the finding that waterlogging resulted in a decrease in total root carbohydrate and activities of starch synthesis-related enzymes, ADP-glucose pyrophosphorylase (AGPase) and starch synthase (Fig. S5), led to the conclusion that *S. cannabina* roots responded to waterlogging by regulating energy consumption and production.

### Effects on carbon metabolism and amino acid metabolism

When plants lack circulatory system to transport oxygen produced by photosynthesis to heterotrophic roots, oxygen-dependent mitochondrial respiration in the root is greatly limited under waterlogging conditions. As verified by qRT-PCR, many genes including well-known hypoxic genes associated with glycolysis and fermentation (ADH and PDC) were induced in the early stage of waterlogging, which indicated that the glycolysis and fermentation pathway was activated to maintain ATP production under stress conditions. As a result, the demand for carbohydrates increased and carbon metabolism significantly increased in waterlogged *S. cannabina* roots. Under hypoxic conditions, the acceleration of carbon metabolism is critical for plant survival^37, 38^. The switch from mitochondrial respiration to fermentation is likely to strongly affect energy and carbon metabolism. The up-regulated expression of genes coding for the glycolytic pathway enzymes under waterlogging treatment can be considered an adaptive response of *S. cannabina* to flooding stress. Consistent with our results, glycolytic enzymes are key factors in the early response to flooding in soybean^39, 40^.

In a comparison of transcriptome responses to low-oxygen in *Arabidopsis*, cotton, and poplar, amino acid metabolism changes were common among the three dicotyledonous species, although there was almost no overlap between specific processes^41^. Waterlogging also led to considerable changes in the levels of amino acids in *S. cannabina* roots (Table S9). In response to the 3 h waterlogging treatment, genes involved in cysteine and methionine metabolism, tyrosine metabolism, beta-alanine metabolism, arginine and proline metabolism, and glycine, serine, and threonine metabolism were upregulated. With the exception of tyrosine, genes involved in the metabolism of these amino acids were also upregulated in the 27 h waterlogging group. Kreuzwieser *et al*. proposed that hypoxia inhibits the TCA cycle and activates the glycolysis and fermentation pathways, resulting in the accumulation of amino acids closely derived from glycolysis intermediates^34^. As seen also in Arabidopsis^42^, most of the accumulating amino acids were closely derived from either pyruvate (e.g. alanine) or intermediates of glycolysis (e.g. glycine, serine, threonine), while the decreasing amino acids (e.g. glutamine, glutamic acid, asparagine, aspartic acid) were mostly derived from TCA cycle intermediates. The block in aerobic respiration caused by waterlogging may led to a decrease in flux into the TCA cycle (due to an accumulation of NADH and depletion of NAD^+^ in the mitochondrial matrix), resulting in a redirection of glycolytic carbon into glycolytic intermediate-derived amino acids and a decrease of flux out of the mitochondria into TCA cycle intermediate-derived amino acids^34^.

### Responses of transcription factors

Currently, the known transcription factors that respond to waterlogging stress are basic leucine zipper (bZIP)^43^, NAC family^44^, WRKY^45^, MYB^46^, ETHYLENE RESPONSE FACTOR (ERF), and basic helix-loop-helix (bHLH)^47, 48^ factors. These transcription factors control the expression of their target genes, which determines the waterlogging tolerance of plants. The MYB transcription factors regulate the expression profiles of a large number of stress-responsive genes, which are the so-called target genes. For instance, overexpression of *OsMYB2*, a rice MYB gene, activates proline synthetase and transporter genes as well as other stress-related genes^49–52^. The present study showed similar findings. In the KOG analysis (Fig. 1), 5.78% (3358) of the total transcripts were predicted to be related to transcription, indicating that waterlogging stress induced high transcriptional activity. Further illustrating the active responses of the transcription factors is under our experimental sets.

## Conclusion

In the present study, high-throughput RNA-sequencing technology was used to analyze differential gene expression profiles of *S. cannabina* roots subjected to short-(3 h) and long- (27 h) term waterlogging stress. The expression of 2719 and 9738 DEGs was reprogrammed by short and long exposure to waterlogging, respectively (Fig. S6). Transcript comparison in *S. cannabina* under short and long term waterlogging conditions using NGS technology shed light on the molecular basis of the responses of this waterlogging-tolerant legume. Under hypoxic stress conditions, the glycolysis and fermentation pathway was activated to produce ATP. A series of energy-consuming biosynthetic processes were dramatically repressed in response to short and long term waterlogging, whereas amino acid metabolism was induced to help maintain ATP. The assembled transcript sequences and comparison analysis with public transcriptome data revealed the key genes and metabolic pathways playing important roles in waterlogging tolerance. The present study provides a foundation for future studies on *S. cannabina* at the cellular and genomic levels.

## Methods

### Plant material and treatments

Seeds of *S. cannabina* (Retz.) *Pers*. (Shandong Academy of Agricultural Sciences, China) from a single population were sterilized in 10% hydrogen peroxide for 10 min and rinsed several times with distilled water before use, then germinated at 28 °C for 48 h and sown (thinned to three uniform seedlings after germination) in a single 2-Liter pot containing autoclaved zonolite, arranged randomly in a greenhouse with day/night temperatures of 30/22 °C, 60% ± 2% relative humidity, and a photoperiod of 14/10 h light/dark. The photon flux density was approximately 800 μmol m^−2^ s^−1^. Hoagland solution was supplied regularly and the pots were weighed every week to adjust the water content. The same conditions were used for the treatments. The pots were placed in watertight containers and filled with room temperature water maintaining a water level above the soil surface as the treatment. Untreated plantlets were placed in the same container as the control. Thirty day-old plantlets were used for waterlogging treatment. The 27 h sample was waterlogged first, after 24 hours, the 3 h sample was treated, when the treatment was all done, all samples from three treatments (0 h, 3 h, and 27 h) for RNA isolation were harvested at same time point, snap frozen in liquid nitrogen and stored at −80 °C.

### RNA isolation, cDNA library construction, and sequencing

Root samples from 3 seedlings were pooled for a single biological replicate; total RNA from 2 biological replicates was isolated using TRIzol (Invitrogen, USA) following the manufacturer’s instructions. RNA was checked on 1% agarose gels to avoid possible degradation and contamination, and was then examined by a Nano Photometer spectrophotometer (IMPLEN, CA, USA) for RNA purity. Qubit RNA Assay Kit in Qubit 2.0 Flurometer (Life Technologies, CA, USA) was used to measure RNA concentration, and RNA Nano 6000 Assay Kit of the Bioanalyzer 2100 system (Agilent Technologies, CA, USA) was used to evaluate RNA integrity. Only RNA samples that passed the quality tests were chosen for RNA-Seq analyses.

RNA-Seq library construction was performed using the NEBNext Ultra Directional RNA Library Prep Kit for Illumina (NEB, Ispawich, USA) following manufacturer’s instructions. Four index codes were added to assign sequences for each sample. In brief, mRNA was purified from 3 μg total RNA using poly-T oligo-attached magnetic beads (Life technologies, CA, USA), and then fragmented using divalent cations under elevated temperature in the NEB proprietary fragmentation buffer. Double-stranded cDNAs were synthesized using random hexamers and M-MuLV Reverse Transcriptase (RNase H^−^), followed by DNA Polymerase I and RNase H. After adenylation of the 3′ ends of DNA fragments, Illumina PE adapter oligonucleotides were ligated to prepare for hybridization. To select cDNA fragments of the preferred 200 bp in length, the library fragments were purified using an AMPure XP system (Beckman Coulter, Beverly, CA, USA). cDNA fragments with ligated adaptor molecules on both ends were selectively enriched using Illumina PCR Primer Cocktail in a 12-cycle PCR reaction. The products were purified (AMPure XP system) and quantified using the Agilent high-sensitivity DNA assay on the Agilent Bioanalyzer 2100 system (Agilent, Santa Clara, CA, USA). The clustering of the index-coded samples was performed on a cBot Cluster Generation System using TruSeq PE Cluster Kit v3-cBot-HS (Illumina) according to the manufacturer’s instructions. RNA-seq libraries were sequenced on an Illumina HiSeq. 4000 platform to generate 150 bp paird-ended (PE) reads. This procedure was carried out by Novogene Bioinformatics Technology Co. Ltd (Tianjing, China).

### *De novo* transcriptome assembly and annotation

Raw reads were pre-processed to remove low quality regions and adapter sequences using NGS QC Toolkit (v2.3)^53^. At the same time, Q20, Q30, GC-content, and the sequence duplication level of the clean data were calculated. All the downstream analyses were based on clean data with high quality. Transcriptome assembly was achieved using Trinity^54^ with min_kmer_cov set to 2 by default and all other parameters set to default. Gene function was annotated based on the following seven databases, Nr (NCBI non-redundant protein sequences), Nt (NCBI non-redundant nucleotide sequences) and Swiss-Prot (a manually annotated and reviewed protein sequence database) using NCBI blast 2.2.28 + software with a cutoff E-value of 10^−5^; KOG/COG (Clusters of Orthologous Groups of proteins) using NCBI blast 2.2.28^+^ software with a cutoff E-value of 10^−3^; KO (KEGG Ortholog database) using KAAS, KEGG Automatic Annotation Server with a cutoff E-value of 10^−10^; Pfam (Protein family) using HMMER 3.0 package with a cutoff E-value of 0.01; and GO (Gene Ontology) annotation was performed by using Blast2GO software with a cutoff E-value of 10^−6^.

### Differential expression analysis

Gene expression levels were estimated by RSEM^55^ for each sample as follows: firstly, clean data were mapped back onto the assembled transcriptome by bowtie2 using default settings (mismatch 0). Then, the read count for each gene was obtained from the mapping results and normalized to fragments per kilo base of transcript sequence per millions base pairs sequenced is recommended for paired-end reads (FPKM). Differential expression analysis of two treatments was performed using the DESeq^56^ R package (1.10.1). The resulting P values were adjusted using the Benjamini’s approach^57^ for controlling the false discovery rate. FDR adjusted P < 0.05 and absolute value of log_2_ ratio >1 were set as the threshold for significance of gene expression differences between treatments. GO enrichment analysis of DEGs was implemented by the GOseq R package based on Wallenius non-central hyper-geometric distribution^58^, which can adjust for gene length bias in DEGs. KOBAS^59^ software was used to test the statistical enrichment of DEGs in KEGG pathways.

### Quantitative real-time PCR

The expression patterns of 12 genes involved in phenylpropanoid biosynthesis, glycolysis pathway, and amino acid metabolism were analyzed using qRT-PCR. Three independent biological replicates of root tissue were used for RNA extraction for qRT-PCR assays. The reverse transcription reactions were carried out by GoScript™ Reverse Transcription System (Promega, USA). Gene-specific primers were designed according to the reference unigene sequences using Primer Premier 5.0 (Table S11). All reactions were performed on an iCycler iQ real-time PCR detection system (BIO-RAD) with three technical replicates. Each reaction was performed in a total volume of 20 μl including 0.2 μM primer pairs, 2 μl diluted cDNA, and 10 μl 2× SYBR Green PCR Master Mix (TaKaRa Bio Inc., Dalian, China). The amplification reactions were incubated at 95 °C for 30 s, followed by 40 cycles of 95 °C for 5 s, 58 °C for 15 s, and 72 °C for 20 s. *S. cannabina* actin and tubulin gene were used as normalizer (Table S11), and relative gene expression levels were calculated using the 2^−△△*C*t^ method.

### Carbohydrate content analysis

Frozen root tissue (100 mg) was used to quantify the total carbohydrate content using a method described by Juntawong *et al*.^1^, with three replicates for each sample. Non-structural carbohydrates were extracted and hydrolyzed by adding 3 ml of 3 M HCl and incubated in a boiling water bath for 3 h. The extract was neutralized by Na_2_CO_3_. The anthrone method was used to determine total carbohydrate content relative to a standard series of glucose. In brief, the extract (200 μl) and distilled water (300 μl) were mixed with 2 ml of 0.14% (w/v) anthrone solution in 95% H_2_SO_4_, incubated in a boiling water bath for 8 min, and rapidly cooled on ice. The absorbance was quantified at 630 nm.

### Assay of activities of the enzymes

The crude extracts of AGPase or starch synthase were prepared according to Nakamura *et al*.^60^ with modifications. In brief, 0.3 g of root tissues was homogenised on ice in a 1 ml pre-cooled solution composed of 100 mM Hepes-NaOH at pH 7.4, 5 mM MgCl_2_, 3 mM EDTA, 12.5% (v/v) glycerol, 5% (v/v) polyvinylpyrrolidone, and 50 mM β-mercaptoethanol. The homogenate was centrifuged at 4 °C at 12,000 × g for 5 min. The supernatant was collected for AGPase or starch synthase activities analysis. For AGPase activity, a 30 μl aliquot of the crude enzyme extract was mixed well with 110 μl reaction solution comprising 100 mM Hepes-NaOH at pH 7.4, 5 mM MgCl_2_, 3 mM pyrophosphoric acid, 4 mM dithiothreitol (DTT) and 1.2 mM adenosine diphosphate glucose (ADPG), incubate in a 30 °C water bath for 20 min, then terminated by treatment for 30 s in a boiling water. The reaction solution was cooled rapidly in an ice-water, and then centrifuged for 10 min at 10,000 × *g* at 4 °C. A 100 μl aliquot of the supernatant was added to 5.2 μl solution composed of 5.76 mM nicotinamide adenine dinucleotide phosphate (NADP), 0.07 U glucose-6-P-dehydrogenase and 0.08 U β-glucomutase, incubate in a 30 °C water bath for 10 min, terminated by treatment for 30 s in a boiling water. A control was conducted in parallel in the reaction system composed of a 20 μl aliquot of the crude enzyme extract and the sterile deionised water to correct the background glucose release. The OD_340 nm_ in the reaction solution were measured and normalized by OD_340 nm_ resulting from the control. The enzyme activity was then estimated with normalized OD_340 nm_ in the reaction solution against the standard concentration curve established at 340 nm with a series of 100 mM Hepes-NaOH solutions with gradient concentrations of NADPH. Each reaction was replicated 3 times.

To examine starch synthase activity, a 20 μl aliquot of the enzyme extract was mixed with 40 μl solution composed of 50 mM Hepes-NaOH at pH 7.4, 15 mM DTT, 0.7 mg amylopectin and 1.6 mM ADPG, incubate 20 min, then in a 100 °C water bath, and cooled rapidly in an ice-water bath. It was further mixed with a 20 μl solution containing 50 mM Hepes-NaOH at pH 7.4, 1.2 U pyruvate kinase, 4 mM phosphoenolpyruvate, 10 mM MgC1_2_ and 200 mM KCl, incubate 20 min, terminated by 100 °C water bath. It was immediately cooled in an ice-water bath, and then centrifuged at 10,000 × *g* at 4 °C for 10 min. A 60 μl aliquot of the supernatant was mixed with 40 μl solution containing 50 mM Hepes-NaOH at pH 7.4, 2 mM NADP, 10 mM glucose, 0.35 U glucose-6-P dehydrogenase, 1.4 U hexokinase and 20 mM MgCl_2_, incubated for 10 min. The starch synthase activity was then determined with the OD_340 nm_ in the reaction solution against a standard concentration curve established with gradient concentrations of NADPH at 340 nm. Each reaction was replicated 3 times.

## Electronic supplementary material

Supplementary Information

Table S1

Table S2

Table S3

Table S4

Table S5

Table S6

Table S7

Table S8

Table S9

Table S10

Table S11
