# Supplementary material for: Elucidation of the molecular responses to waterlogging in Sesbania cannabina roots by transcriptome profiling
Source: Sci Rep. 2017 Aug 23;7:9256. doi: 10.1038/s41598-017-07740-5 (PMC5569044; doi:10.1038/s41598-017-07740-5)
Supplement: Supplementary file 1 — Supplementary Information [file 41598_2017_7740_MOESM1_ESM.pdf]

## Supplementary files:

Elucidation of the molecular responses to waterlogging in *Sesbania cannabina* roots by transcriptome profiling

**Authors:**

Cheng-Gang Ren, Cun-Cui Kong, Kun Yan, Hua Zhang, Zhi-Hong Xie

Corresponding author: Zhi-Hong Xie

Email: zhxie@yic.ac.cn

Telephone: +86-0535-2109183

## Supplementary figures:

**Supplementary figure S1 Cluster analysis of the transcriptome of *S. cannabina* plantlets subjected to different waterlogging conditions**

Each column represents a sample and each line represents a single gene. Different colors indicate different expression levels. Red indicates upregulation and green indicates downregulation, whereas black indicates unchanged expression.

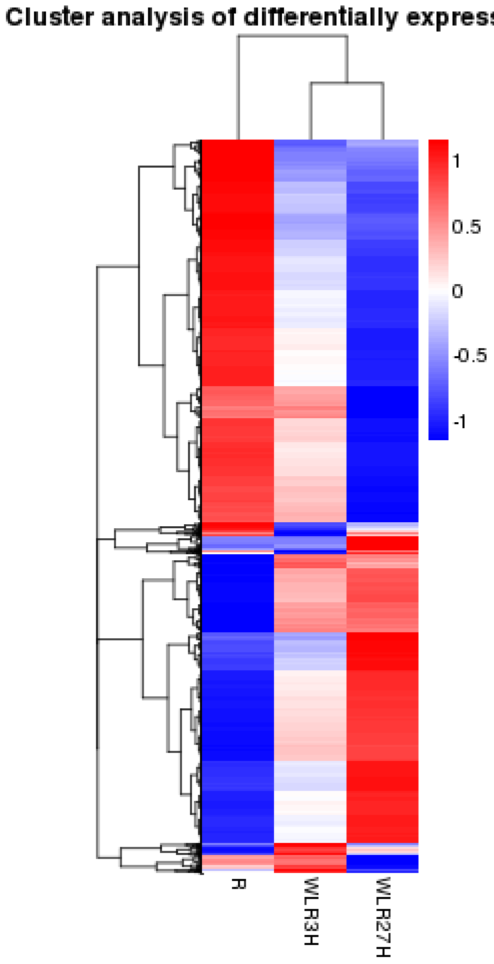


**Supplementary figure S2: Unigenes predicted to be involved in the phenylpropanoid biosynthesis pathway derived from KEGG database [61].** Red outline indicates significantly increased expression in waterlogging treatment; green outline indicates significantly decreased expression; yellow outline indicates proteins encoded by both up-and down-regulated genes.

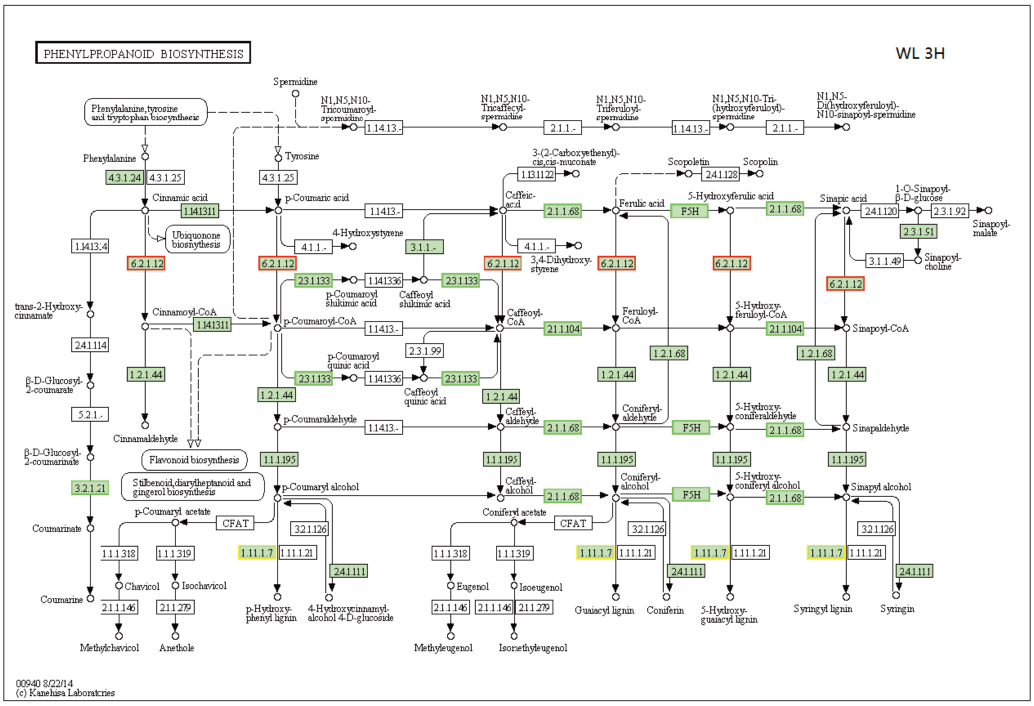


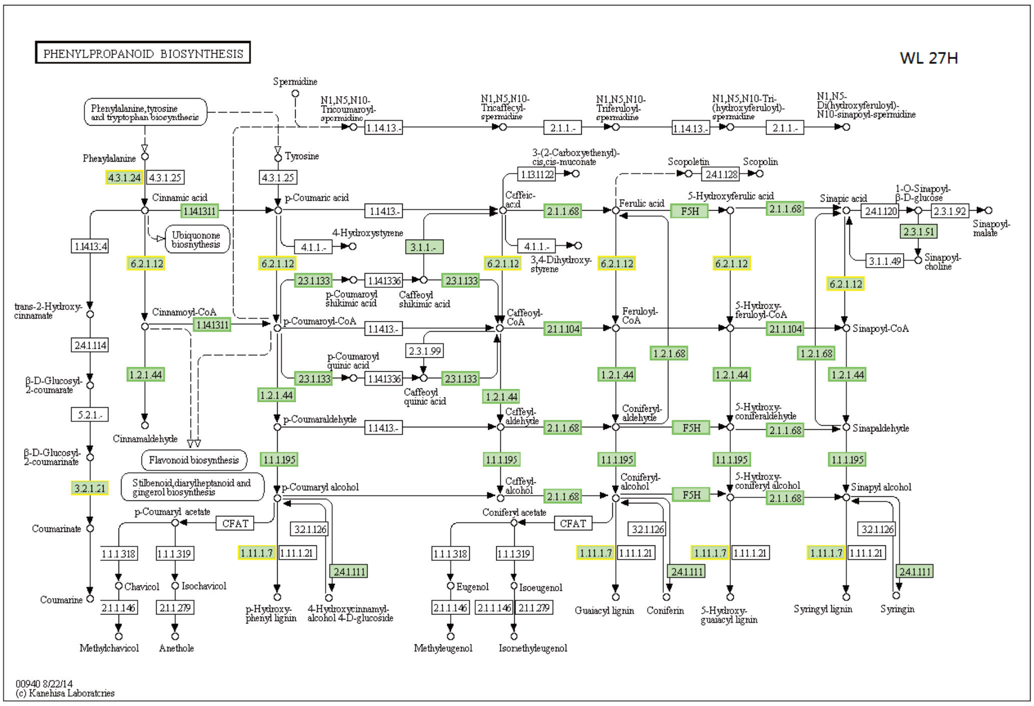


**Supplementary figure S3. Real-time PCR validations of selected DEGs during waterlogging**

**A**, The relative expression of DEGs between 0 h and 3 h waterlogging treatment; **B**, The relative expression of DEGs between 0 h and 27 h waterlogging treatment. Relative expression was analyzed by the $2^{-\Delta\Delta CT}$ method. The results of qRT-PCR are

expressed as the mean ±standard deviation (±SD) of at least three replicates. (Cluster-8728.18814 was annotated as 4-coumarate--CoA ligase, 4CL, Cluster-8728.82713 as peroxidase, POD, Cluster-8728.113801 as glucose-6-phosphate isomerase, GPI, Cluster-8728.125377 as pyruvate kinase, PDC, Cluster-8728.59970 as alcohol dehydrogenase, ADH, Cluster-8728.74364 as aspartokinase-homoserine dehydrogenase, AK-HSDH, Cluster-8728.89282 as copper amine oxidase, CuAO1, Cluster-8728.82384 as polyamine oxidase, PAO, Cluster-8728.15515 as aspartate aminotransferase, AAT, Cluster-8728.90878 as alanine aminotransferase 1, ALAAT1, Cluster-8728.92583 as WRKY transcriptional factor 20, WRKY20, and Cluster-8728.104232 as NAD(P)H dehydrogenase, QR).

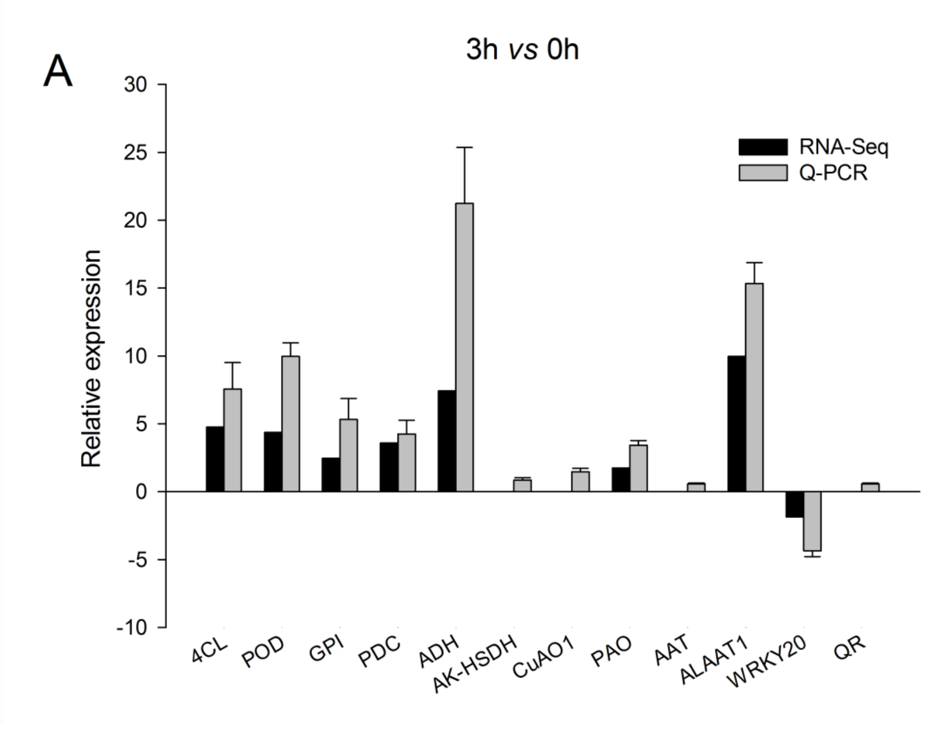

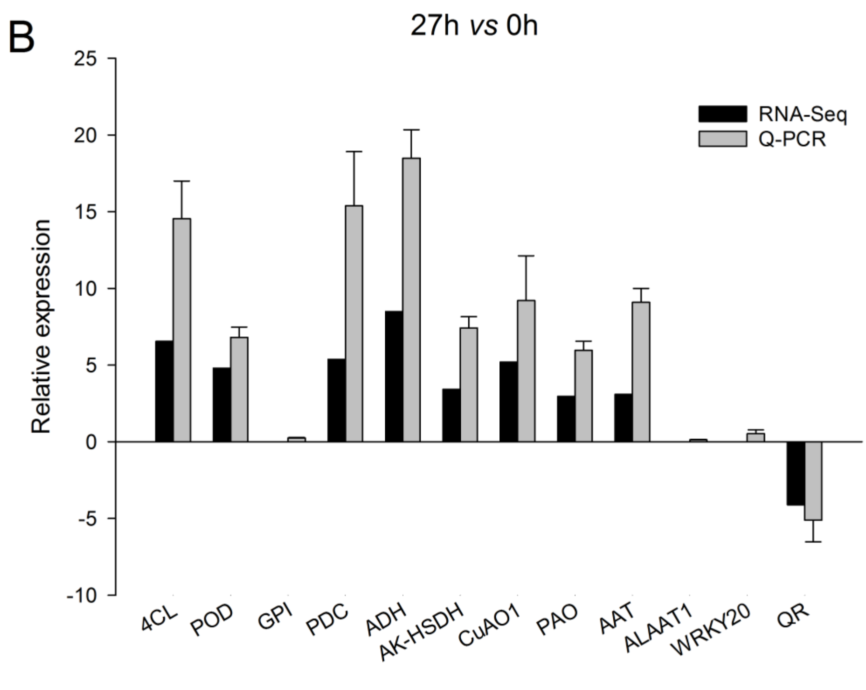


**Supplementary figure S4: Comparison of expression profile by RNA-Seq and qRT-PCR.** Comparison of expression profiles of 12 selected genes by RNA-Seq and RT-qPCR showing different expression profiles. Each data point represent the log2 normalized expression level obtained from RNA-Seq (y axis) and qRT-PCR (x axis) analyses. S. cannabina actin and tubulin gene were used as normalizer.

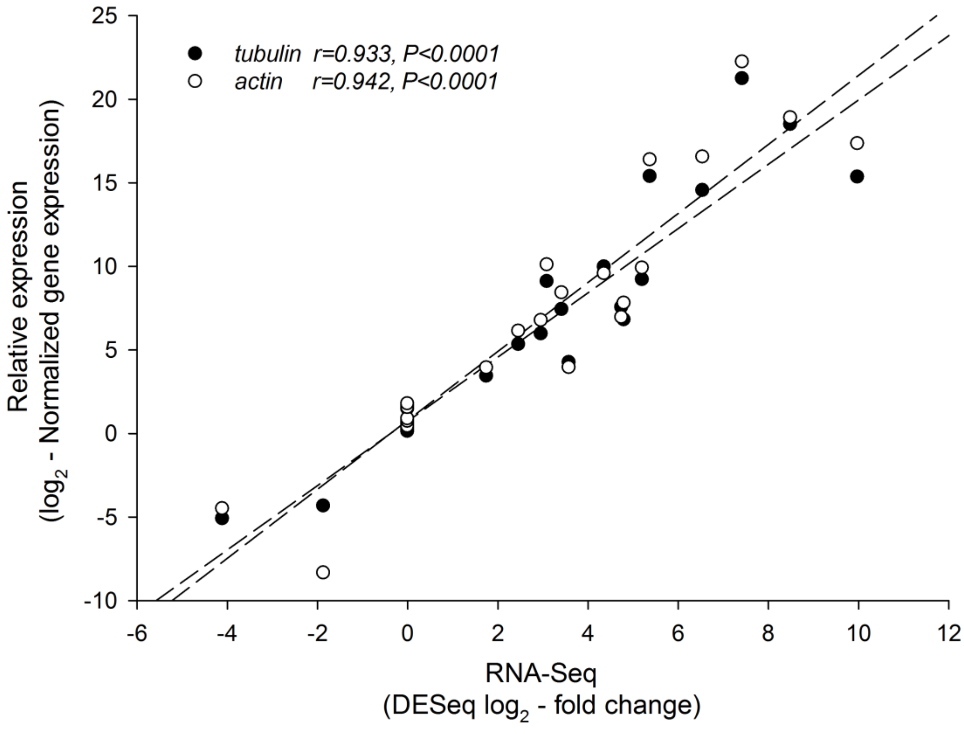

**Supplementary figure S5: Total root carbohydrate content and enzymes activities after waterlogging. A,** Total root carbohydrate content. **B,** The activity of AGPase. **C,** The activity of starch synthase. Bars with different letters among times are significantly different (one-way ANOVA, Duncan's multiple range test, $P < 0.05$).

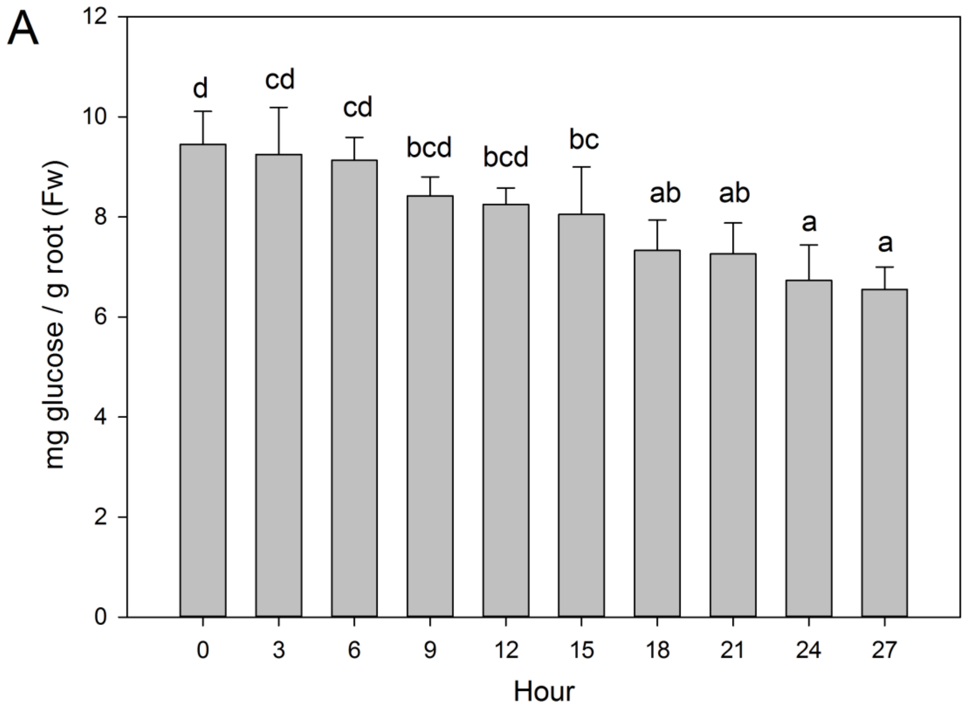


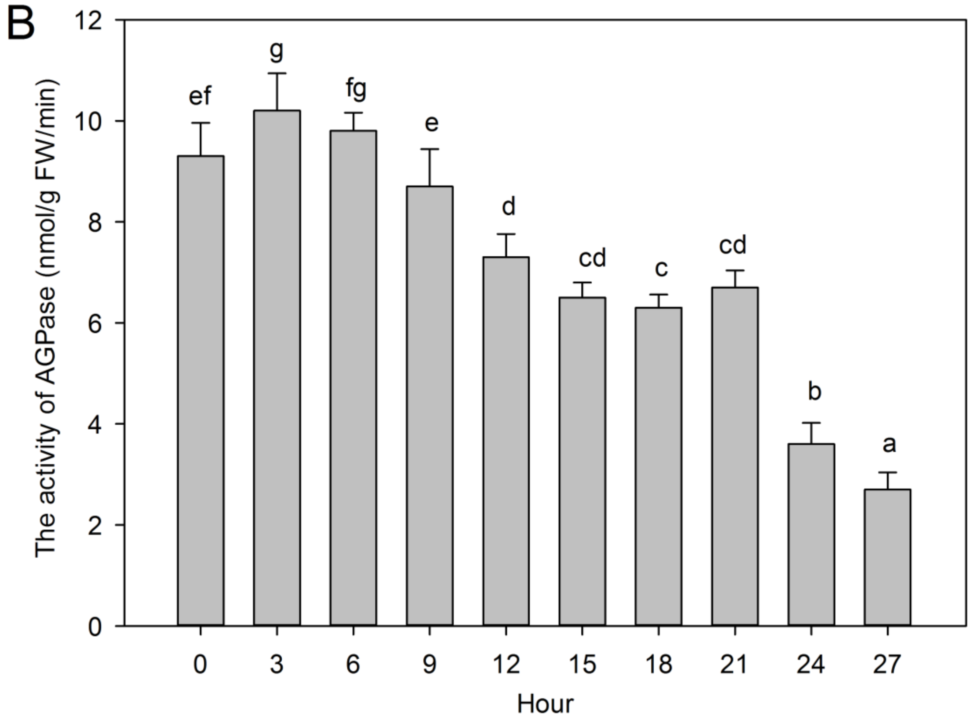

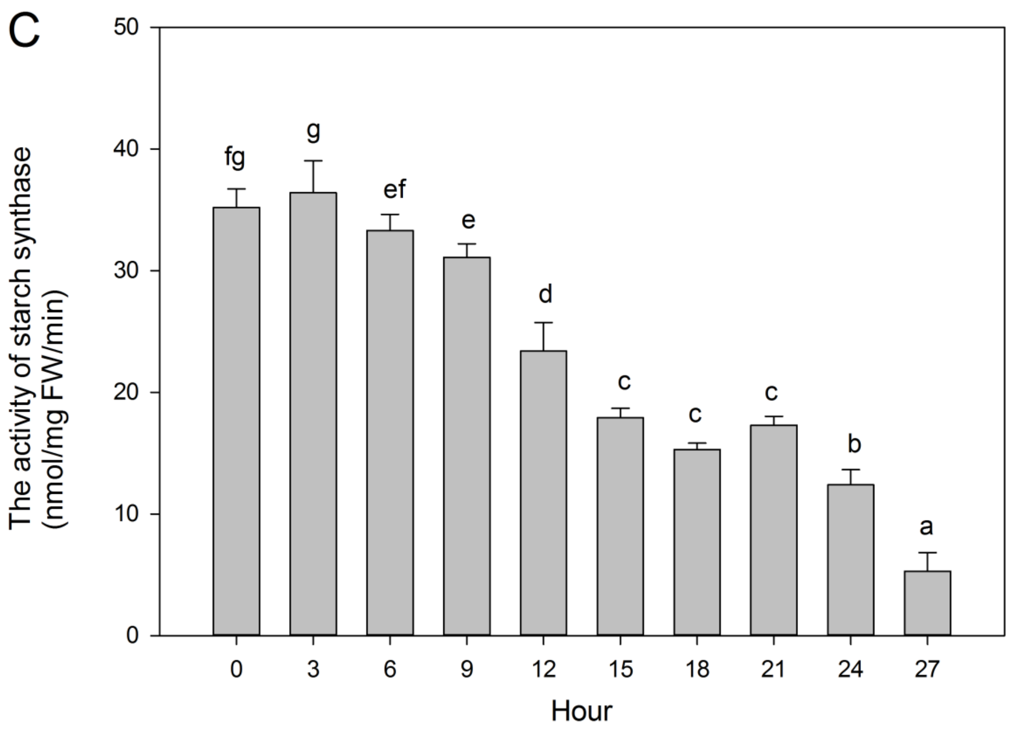


**Supplementary figure S6: Venn diagrams of the differential expression transcripts under 3 and 27 hour waterlogging treatment in *S. cannabina* plantlets.**

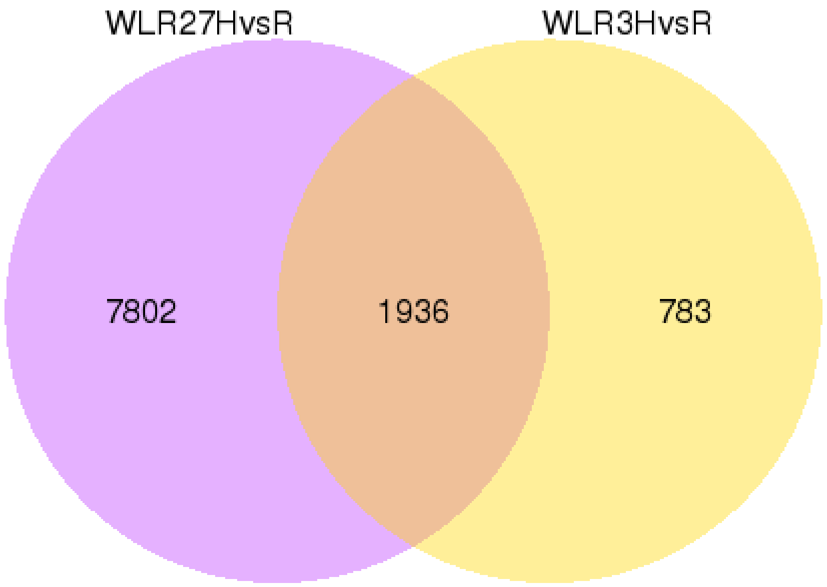


## Supplementary tables:

**Supplementary table S1: Length frequency distribution and length distribution**

**of transcript and unigene after assembly.**

**Supplementary table S2: DEGs readcount analysis of each treatment** (See Excel file)**.**

**Supplementary table S3: GO enrichment of up-regulated DEGs in 3h waterlogging treatment** (See Excel file)**.**

**Supplementary table S4: GO enrichment of down-regulated DEGs 3h waterlogging treatment** (See Excel file)**.**

**Supplementary table S5: GO enrichment of down-regulated DEGs in 27h waterlogging treatment** (See Excel file)**.**

**Supplementary table S6: GO enrichment of up-regulated DEGs in 27h waterlogging treatment** (See Excel file)**.**

**Supplementary table S7: KEGG pathway enrichment of DEGs in 3h waterlogging treatment** (See Excel file)**.**

**Supplementary table S8: KEGG pathway enrichment of DEGs in 27h waterlogging treatment** (See Excel file)**.**

**Supplementary table S9: KEGG pathway enrichment of up- and down-regulated DEGs in waterlogging treatment** (See Excel file)**.**

**Supplementary table S10: Details of differentially expressed TFs identified by RNA-Seq in waterlogging treatment** (See Excel file)**.**

**Supplementary table S11: Real-time PCR primer information** (See Excel file)**.**
